# Calcineurin regulates septin cytoskeleton organization in the human fungal pathogen *Aspergillus fumigatus*

**DOI:** 10.1016/j.bbrc.2026.154773

**Published:** 2026-10-19

**Authors:** D. Christopher Cole, Erik J. Soderblom, William J. Steinbach, Praveen R. Juvvadi

**Affiliations:** a Department of Pediatrics, Duke University School of Medicine, Durham, NC, USA; b Proteomics and Metabolomics Core Facility, Duke University School of Medicine, Durham, NC, USA; c Department of Pediatrics, University of Arkansas for Medical Sciences, Little Rock, AR, USA; d Arkansas Children’s Research Institute, Little Rock, AR, USA

**Keywords:** *Aspergillus fumigatus*, Calcineurin, Septins, Actin, Tubulin

## Abstract

Signaling events leading to fungal growth and septation depend on key cytoskeletal proteins, including actin, microtubules, and septin GTPases, that assemble into oligomeric structures and filaments and associate with cellular membranes. We previously demonstrated that deleting calcineurin, a calmodulin-dependent protein phosphatase in *Aspergillus fumigatus*, results in stunted hyphal growth, hyperbranching, and aberrant septation. Supporting this, we found that calcineurin colocalizes with actin during early septation, and its localization and activity at mature septa are essential for regular septation, hyphal extension, and growth. However, calcineurin’s interactions and functions at the septa remain unknown. In this study, using GFP-Trap^®^ chromatography and mass spectrometry, we show that calcineurin interacts with actin, tubulin, and septins. To better understand the significance of calcineurin-septin interactions for hyphal extension and septation, we used fluorescence microscopy to visualize septins (AspA~E) in the *A. fumigatus* calcineurin double deletion mutant (Δ*cnaA* Δ*cnaB*). Strikingly, all septins exhibited aberrant localization patterns in the calcineurin mutant. Specifically, AspA mislocalized from punctate structures in the cytoplasm, AspB localized to longer rod-like structures, AspC mislocalized from the septa, AspD mislocalized from tubular and ring-like structures, and AspE mislocalized from long filaments to punctate structures resembling microtubule-organizing centers (MTOCs). Treatment with the calcineurin-specific inhibitor FK506 also caused AspE to mislocalize from filaments to MTOCs, a pattern mimicking the treatment with microtubule inhibitor nocodazole. These results highlight the unique importance of calcineurin for complete septin organization, which is crucial for proper septation and ultimately hyphal growth.

## 1. Introduction

The human fungal pathogen *Aspergillus fumigatus* causes deadly invasive aspergillosis disease in immunocompromised patients and is categorized as a “critical priority pathogen” by the WHO [1]. Invasive hyphal growth and host tissue invasion are critical to *A. fumigatus* pathogenesis. This process is directly facilitated by dynamic hyphal tip extension and formation of unique septal compartments to maintain hyphal rigidity and integrity [2].

We previously reported that the calcineurin complex, also known as calmodulin-dependent protein phosphatase, is essential for *A. fumigatus* hyphal growth, septation, and invasive disease [3,4]. The *A. fumigatus* calcineurin complex is comprised of a catalytic subunit (CnaA) and a regulatory subunit (CnaB) [5]. The deletion of the calcineurin complex resulted in a severe hyperbranching phenotype with stunted hyphae, aberrant septation, defects in the cell wall and virulence [3]. Supporting this, we demonstrated that the *A. fumigatus* calcineurin complex regulates hyphal extension by localizing as punctate vesicular structures in actively growing hyphal tips and septa [3,6]. We also found that mislocalization of the calcineurin complex from the septum, whether due to mutations or chemical inhibition by the calcineurin-specific inhibitor FK506, causes aberrant septation and blocks hyphal extension [7,8]. We further confirmed that defects in septation and hyphal extension are not mediated via the calcineurin-dependent transcription factor CrzA or the major calcineurin-binding protein CbpA, as the deletion of *crzA* (Δ*crzA*) or *cbpA* (Δ*cbpA*) did not impact septation [9,10]. These studies led us to focus on calcineurin’s functional interactions with as-yet-unknown effectors at the active points of growth to directly control hyphal septation, extension, and ultimately pathogenesis. Given that calcineurin co-localized with actin during early septation process and remained localized at mature septa [3], we reasoned that calcineurin interactions with cytoskeletal components at the septa might be important for proper hyphal extension and regular septation.

Using a CnaA-GFP expressing strain, we performed GFP-Trap^®^ affinity purification followed by mass spectrometry. For the first time, we identified key cytoskeletal proteins, including actin, tubulin, and the major core septins AspA, AspC, and AspD, as calcineurin interactors. While the interactions between the septins and actin cytoskeleton are reported [11,12], and studies also have shown their interaction with microtubules [13,14], we were particularly interested in examining the link between septins and calcineurin. We earlier reported the different localization patterns of four core septins (AspA~D), and a filamentous fungal-specific septin, AspE, in *A. fumigatus* [15]. In this study, we expressed individual septins in a calcineurin double deletion mutant background. We reveal that each septin exhibits different and aberrant localization patterns in comparison to the wild-type strain, indicating a novel role for calcineurin in septin organization.

## 2. Materials and methods

### 2.1. Strains construction, culturing, and fluorescence microscopy

To localize AspA, AspB, AspD, and AspE septins the respective cDNAs cloned individually at the N-terminus of *egfp* in the pUCGH plasmid, and the *AspC* cDNA cloned at the C-terminus of *mcherry* in the plasmid pUCnCrH harboring the hygromycin resistance gene were utilized [15]. The sequenced plasmids were transformed individually into the *A. fumigatus* calcineurin double mutant (Δ*cnaA* Δ*cnaB*). The hygromycin B marker resistant transformants were selected and cultured as previously described [3]. Wild-type (AF293) strains individually expressing all five septins have been reported previously [15]. For all assays, *A. fumigatus* AF293 (wild-type) and *A. fumigatus* calcineurin deletion strains expressing individual septins (AspA~E) were used. These strains were grown in glucose minimal medium (GMM) for 18–36 h at 37°C for microscopy. Fluorescence microscopy for the visualization of septins was performed as previously described [3]. To investigate the effect of FK506 on AspE localization, 18 h control cultures were treated with FK506 (50–100 ng/ml) for 2 h.

### 2.2. GFP-Trap^®^ chromatography and mass spectrometry

The *A. fumigatus* strain expressing CnaA-GFP was cultured in biological triplicates in GMM liquid medium for 24 h (250 rpm; 37°C). Approximately 2 g wet weight mycelia were thoroughly washed and homogenized in 5 ml lysis buffer (10 mM Tris-HCl pH 7.5, 150 mM NaCl, 0.5 mM EDTA, 0.01% Triton X-100, 1 mM DTT, 1 mM PMSF, and 1:100 Protease Inhibitory Cocktail (Sigma Aldrich)) using liquid nitrogen as previously reported [16]. Briefly, the homogenized suspension was centrifuged twice (5000 rpm for 10 min; 7000 rpm for 15 min at 4°C) to obtain clarified crude extract, protein was quantified by the Bradford method and normalized to approximately 10 mg protein per 5 ml prior to GFP-Trap^®^ chromatography (Chromotek). Next GFP-Trap^®^ agarose purification, Rapigest SF surfactant supplementation, reduction of samples in dithiothreitol followed by alkylation with iodoacetamide, and protein digestion with trypsin for mass spectrometry was performed according to the protocol previously described [16,17].

For quantitative LC/MS/MS 1 μl of each sample was purified on a nanoAcquity UPLC system (Waters Corp) coupled to a Thermo QExactive Plus high-resolution mass tandem spectrometer via a nanoelectrospray ionization source. The sample trapped on a symmetry C18 trapping column (300 mm × 180 mm; flow-rate 5 μl/min; 99.9/0.1 v/v water/acetonitrile) was separated on a 1.7 μm Acquity HSS T3 C18 column (75 mm × 250 mm; 90-min linear gradient; 5% to 40% acetonitrile containing 0.1% formic acid; flow rate of 400 nl/min; column temperature 55°C). Data was collected on the QExactive Plus mass spectrometer in data-dependent acquisition (DDA) mode. A full scan (r = 70,000 @ *m*/*z* 200) performed from *m*/*z* 375 – 1600 with a target AGC value of 1e6 ions was followed by 10 MS/MS scans (r = 17,500 @ *m*/*z* 200) at a target AGC value of 5e4 ions. To increase depth of coverage a 20s dynamic exclusion was employed. For each sample injection the total analysis cycle time was approximately 2 h. LC-MS/MS data was imported into Rosetta Elucidator v3.3 (Rosetta Biosoftware, Inc) and aligned using the PeakTeller algorithm (Elucidator) based on the accurate mass and retention time of detected ions that contained MS/MS spectra. Relative peptide abundance was calculated based on the area-under-the-curve (AUC) of aligned features across all runs. Protein abundances were calculated by summing all peptides identified for each protein. MS/MS data were searched against a custom NCBI_RefSeq_Aspergillus database containing yeast alcohol dehydrogenase sequences. Database searching performed on Mascot Server v2.5 (Matrix Science) were annotated at a Mascot ion score of 20 resulting in protein false discovery rate of 4.6% and a peptide false discovery rate of 1.1%.

## 3. Results and discussion

### 3.1. Calcineurin interacts with major cytoskeletal machinery related proteins

We previously established that the calcineurin whole subunit complex, comprising the catalytic (CnaA) and the regulatory (CnaB) subunits, is indispensable for hyphal extension and regular septation in *A. fumigatus* [15]. Furthermore, we demonstrated that the calcineurin complex localizes in punctate dot-like structures that exhibit dynamic retrograde movement from the hyphal tips to sites of early septation. These structures colocalize with actin contractile ring during early stage of septum formation. Interestingly, even after the actin ring diffused following septation, the calcineurin complex remains at the mature septa, indicating calcineurin’s requirement at both the early and later stages of septation. We also confirmed that the defective septation observed in the Δ*cnaA* Δ*cnaB* strain was not due to defect in actin organization [3]. This led to our hypothesis that calcineurin may interact with other key cytoskeletal components to regulate proper hyphal extension and septum formation.

To interrogate if calcineurin interacts with cytoskeletal proteins *in vivo*, we utilized the *A. fumigatus* CnaA–EGFP expressing strain and purified calcineurin interactors by GFP-Trap^®^ affinity purification. Bound proteins were proteolytically digested and analyzed by LC-MS. As shown in Table 1, we identified a total of nine major cytoskeletal proteins, grouped into three categories: actin-related, tubulin-related, and septum-related cytoskeletal GTPases (septins). Actin-related proteins included the major protein Act1 and actin-related proteins VIP1 (actin cytoskeleton protein) and Pan1 (actin cortical assembly protein). The identification of Act1 as a calcineurin interactor supports our previous findings of calcineurin co-localizing with the actin contractile ring during early septation. The second and third groups of proteins included tubulins (tubulin beta, tubulin alpha-1, and tubulin subunit TubB) and three major cytoskeletal septin GTPases (AspA, AspC, and AspD).

Earlier research on septins in mammals highlighted the significance of actin and microtubules for septin organization [12,14,18]. Microtubules are also involved in vesicular transport in filamentous fungi [19]. Our prior work demonstrated that the microtubule inhibitor nocodazole did not affect calcineurin localization at the septum [6].

### 3.2. Core septins exhibit aberrant localization patterns in the calcineurin mutant

Septins belong to a family of well conserved GTP-binding proteins known to regulate morphogenetic processes such as hyphal extension, branching, septation, and conidiation in filamentous fungi [20]. While mammals contain 12 genes (SEPT1–12) [21], filamentous fungi, including *Aspergilli* contain four core septins (AspA~D) and a unique non-core septin (AspE) [15,22]. We previously reported the differential localization patterns of septins in *A. fumigatus* and showed that the filamentous fungal-specific septin, AspE, localized as long filament-like structures that were disrupted by the actin and microtubule inhibitors cytochalasin A and nocodazole, respectively [29]. To gain insight into the significance of the observed interactions of calcineurin with septin GTPases (AspA, AspC, and AspD) which are known to function in the septation process, we localized the various septins by tagging them with EGFP/mCherry fluorescent markers in the background of calcineurin complex deletion. The expression of septins tagged to either EGFP or mCherry did not impact the growth phenotype in either the wild-type or the calcineurin deletion strain (Supplementary Fig. 1).

In the wild-type strain, AspA-GFP localized to discrete punctate structures and throughout the cytoplasm, with no localization observed at the septa (Fig. 1A). However, in the calcineurin deletion mutant, these punctate structures were completely absent, and AspA was distributed only throughout the cytosol (Fig. 1B). Our previous study on calcineurin localization revealed similar punctate structures that dynamically moved from hyphal tips to septation sites [3,6]. Additionally, calcineurin deletion resulted in an irregular and hyperseptated phenotype with shortened hyphal compartments [3]. In contrast, we noted that AspA deletion resulted in significantly extended hyphal compartments compared to the wild-type strain [22], indicating delayed septation in the absence of AspA. Based on these observations, we speculate that calcineurin and AspA may interact within these punctate structures functioning in vesicle trafficking to septation sites and regulating proper septation.

In the wild-type strain, AspB-GFP displayed several ring-like and small doublet rod-like structures at the septa and within the hyphal compartments (Fig. 1C). Conversely, the calcineurin deletion mutant only exhibited long doublet rod-like structures, with no septal localization. These structures occasionally extend throughout the short hyphal compartment (Fig. 1D), suggesting a potential issue with the self-assembly or organization of AspB septin structures in the absence of calcineurin. In our previous study, we showed that AspB deletion also resulted in extended hyphal compartments [22], and AspB interacted with the calcineurin A catalytic subunit [17]. Together, these findings point to a potential role for calcineurin in the assembly of the AspB septin scaffold.

Expression of mCherry-AspC in the wild-type strain showed its localization at the septa and throughout the cytoplasm (Fig. 2A). However, in the calcineurin deletion mutant, AspC was not localized at the septa; instead, it was found in diffused structures within the cytoplasm (Fig. 2B). This indicates that calcineurin is required for the septal localization of AspC.

The expression of AspD-GFP in the wild-type strain revealed its localization in disconnected tubules and ring-like structures at hyphal tips and mature septa (Fig. 2C). In the calcineurin deletion mutant, AspD remained localized at the septa but was completely mislocalized from the tubular and ring-like structures (Fig. 2D). These results indicate that calcineurin is not essential for AspD localization at the septa but is required for the formation of higher-order septin structures in the hyphal tips. Interestingly, unlike other core septins (AspA, AspB, and AspC), AspD is not required for regulating hyphal compartment length [22].

### 3.3. Filamentous fungal-specific septin, AspE, is mislocalized to microtubule organizing centers in the calcineurin mutant

We previously found that, in contrast to the core septins (AspA~D), the filamentous fungal-specific septin AspE is localized to long filament-like structures along the hyphae [15]. As shown in Fig. 3A, in the wild-type strain, AspE localized to hyphal tips and as connected filaments throughout the hyphae, resembling a microtubule network. However, in the calcineurin deletion mutant, AspE-GFP expression resulted in complete mislocalization of AspE from filaments to dot-like structures resembling Microtubule Organizing Centers (MTOCs). We have also shown that AspE interacts with microtubular network [16], which further lends support to this observation. While these MTOCs were primarily present closer to the hyphal tips in actively growing hyphae (Fig. 3B), some MTOCs were found near the septa or in sub-apical regions during early germination (Fig. 3C). This localization pattern mimicked the effect of microtubule destabilizing drugs (nocodazole or benomyl), confirming the nature of these structures as MTOCs. MTOCs are nucleation centers for microtubules at the hyphal tips and the septa [23], which further supports our findings. Next, to confirm if chemical inhibition of calcineurin would yield similar results, we treated the wild-type strain expressing AspE-GFP with FK506, a calcineurin-specific inhibitor. As shown in Fig. 3D, FK506 treatment led to complete disruption of septin filaments and the localization of AspE to MTOCs all throughout the hyphae, confirming the mislocalization observed in the calcineurin deletion mutant.

## 4. Conclusions

This study reveals that calcineurin interacts with key cytoskeletal proteins, an interaction critical for the proper localization of septins. Given that septins rely on actin and microtubules for their assembly and transport, calcineurin’s interaction with these cytoskeletal components may also contribute to the assembly/organization and function of septin complexes. Considering the severe septation defects observed in the calcineurin mutant, and the delayed septation leading to extended apical and subapical hyphal compartments in most septin deletion mutants, we expect that calcineurin-septin interactions are crucial for the precise timing of septation. As calcineurin possesses protein phosphatase activity that regulates the dephosphorylation of specific substrates, further mechanistic studies into the phosphorylation/dephosphorylation of septin complexes in the calcineurin deletion background may unravel calcineurin-dependent septin functions and their inter-relationship with actin and microtubules.

## Supplementary Material

Chris et al-Supplemental Material

## Figures and Tables

**Fig. 1.**
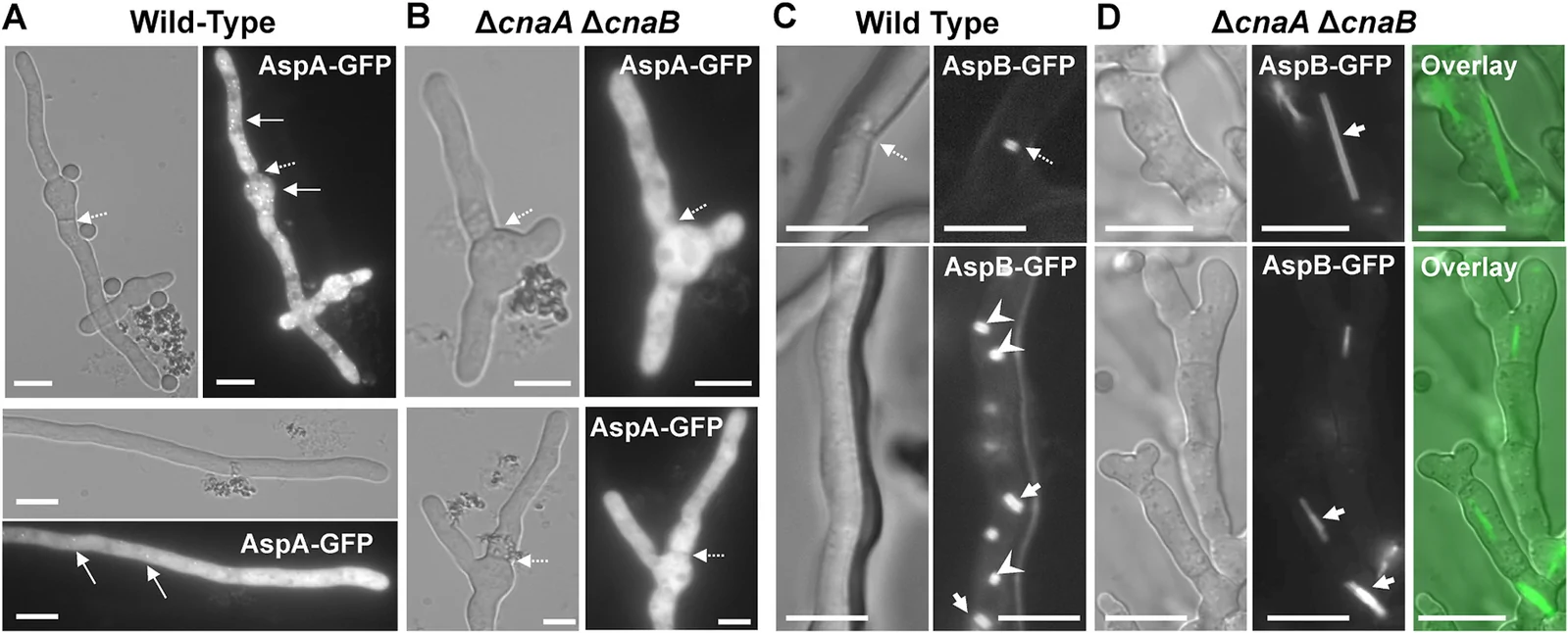
Localization of septins AspA and AspB in *A. fumigatus*. **(A, B)** Localization of AspA-GFP in the germling and hyphal tips in the wild-type and the calcineurin deletion mutant (Δ*cnaA* Δ*cnaB*) strains. Solid arrows indicate punctate dot-like structures within the hyphal compartments and dashed arrows indicate septa. Note the complete mislocalization of AspA to the cytoplasm in the calcineurin deletion mutant strain. **(C, D)** Localization of AspB-GFP is seen as doublet rod like structures at the septa and in ring-like and rod-like structures in sub-apical compartments in the wild-type. The calcineurin mutant showed elongated rod-like structures and complete disappearance of small rod-like and ring-like structures. Dashed arrows indicate septa. Solid arrows indicate doublet rod-like structures, and arrow heads indicate ring-like structures. Scale bar, 10 μm.

**Fig. 2.**
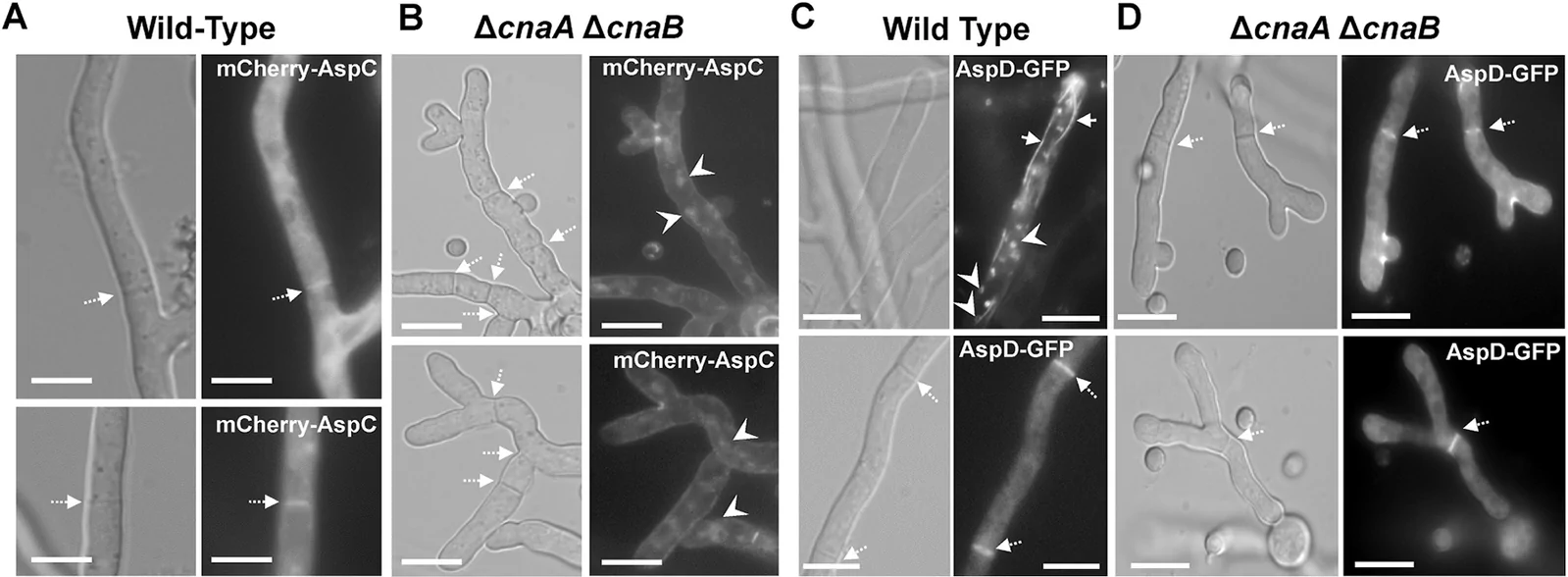
Localization of septins AspC and AspD in *A. fumigatus*. **(A, B)** mCherry-AspC shows septal and cytosolic localization in the wild-type strain but is mislocalized from septa and is in diffuse structures within the hyphal compartments in the calcineurin deletion mutant strain. Dashed arrows indicate septa and arrow heads show diffused localization of AspC in the calcineurin deletion mutant strain. **(C, D)** Localization of AspD-GFP is seen in tubular and ring-like structures in the hyphal tips and is localized at the septa in the wild-type strain. In the calcineurin deletion strain AspD is only seen in the cytoplasm and at the septa. Dashed arrows indicate septa. Solid arrows indicate tubular structures, and arrow heads indicate ring-like structures in the wild-type strain. Scale bar, 10 μm.

**Fig. 3.**
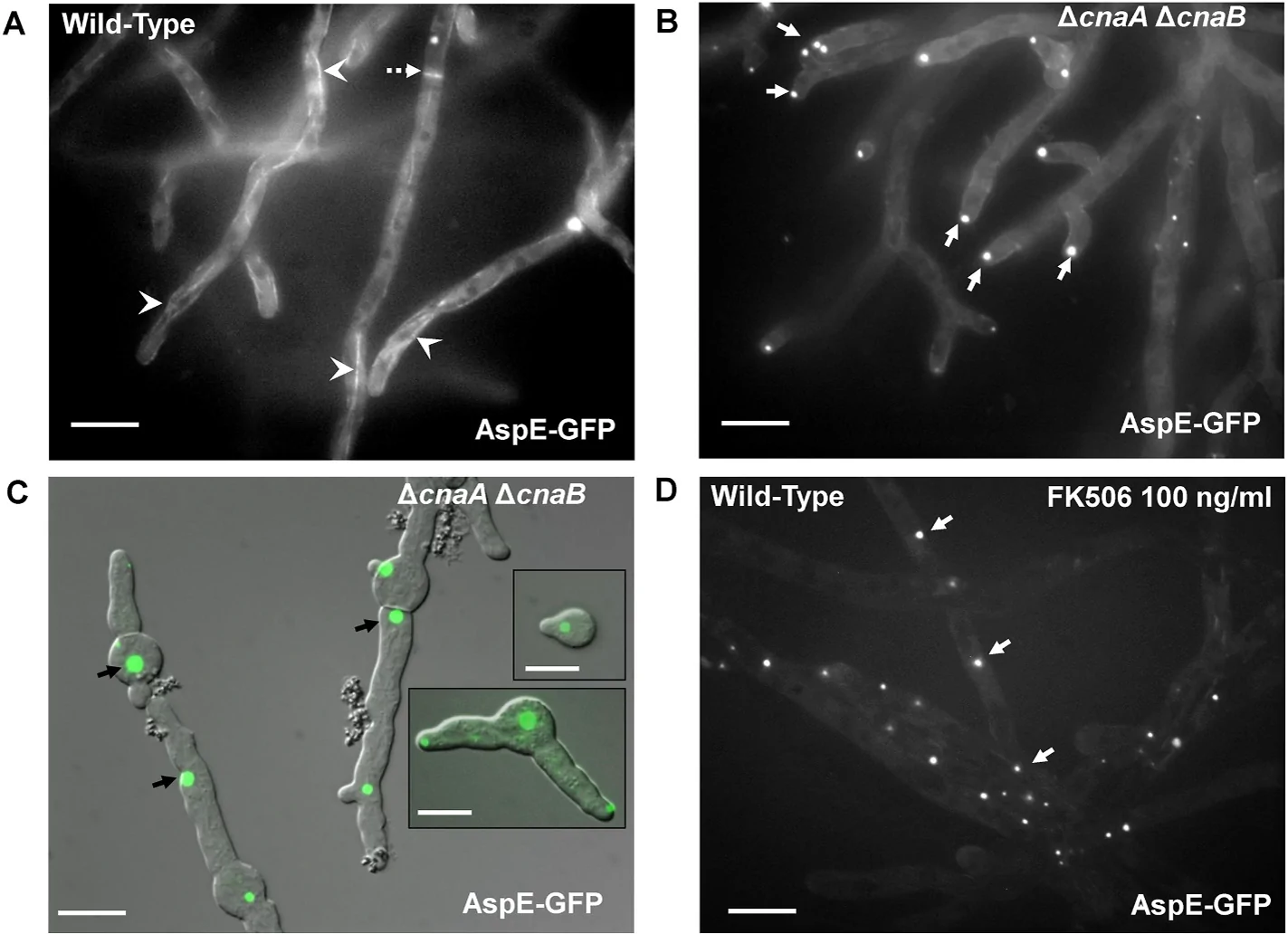
Localization of filamentous fungal-specific septin AspE in *A. fumigatus*. **(A)** AspE-GFP is seen localized as long filaments in the hyphae and also at the septa in the wild-type strain. **(B)** In the calcineurin deletion mutant strain AspE is mislocalized from the filaments to dot-like MTOCs at the hyphal tips. **(C)** During the early phase of germination AspE is seen localized to MTOCs near the germling tips (inset images) and at the septa in the calcineurin deletion mutant strain. **(D)** In the presence of calcineurin specific-inhibitor FK506 AspE is mislocalized from the filaments to the MTOCs. Dashed arrow indicates septum and arrow heads show filaments of AspE in the wild-type strain. Solid arrows indicate MTOCs, black arrows indicate MTOCs localized near the septa in the calcineurin deletion mutant strain. Scale bar, 10 μm.

**Table 1:** Major cytoskeletal proteins identified as calcineurin interactors in *Aspergillus fumigatus*.

Protein	NCBI Accession number	Molecular weight (kDa)	Total Spectral Counts	Uniquely Identified peptides

Septin A (AspA)	XP_753783.1	40	2	VNVIPVIGK
Septin C (AspC)	XP_748035.1	44	8	LTVIDPGFGDYVNNR; VNLIPVIAK; ADTLSPTDLAR
Septin D (AspD)	XP_752252.1	39	7	STLINTIFASHLIDSK; LNIVDTPGYGDQVNNDR LSDVVNVVPVIAK; IKEEFAFHNLK
Actin Act1	XP_747604.1	44	326	HHGIMIGMGQK; DSYVGDEAQSK; RGILTLR
Actin cytoskeleton protein (VIP1)	XP_755336.1	28	26	TTVHVSGISSATTDK; DFFSFCGK; ITSISVTPVSGEK SATVTFEK; FTSALANFDQK; GLHSYFEK; ALDTPSGR; QTEDAPAAAGAEGANVAPSAAPTSQPAGAAAPAEPK
Actin cortical patch assembly protein (Pan1)	XP_749121.1	159	2	SLLSQDAESR
Tubulin beta	XP_752456.1	52	182	AVLVDLEPGTMDAVR; AGPFGELFRPDNFVFGQSGAGNNWAK; GHYTEGAELVDQVIDVVR
Tubulin alpha-1 subunit	XP_750005.1	50	169	EVISLNVGQAGCQIANSCWELYCLEHGIQPDGYLTEER; KKADPDHGFSTFFSETGQGK
Tubulin subunit TubB	XP_755832.1	50	83	LDPEVPESHNNGGSFETFFTETNSGK; SIFVDLDPSPIDEIR; QLFHPELLISGK; EDAANNYAR GHYTVGK; ELVDSVVDR; LSTEYGK; SKLEFSIYPSPR

## Appendix A. Supplementary data

Supplementary data to this article can be found online at https://doi.org/10.1016/j.bbrc.2026.154773.

## References

[1] Morrissey CO, Kim HY, Duong TN, Moran E, Alastruey-Izquierdo A, Denning DW, Perfect JR, Nucci M, Chakrabarti A, Rickerts V, Chiller TM, Wahyuningsih R, Hamers RL, Cassini A, Gigante V, Sati H, Alffenaar JW, Beardsley J, *Aspergillus fumigatus*-a systematic review to inform the World Health Organization priority list of fungal pathogens, Med. Mycol 62 (2024), 10.1093/mmy/myad129. PMC11210617 38935907

[2] Sugui JA, Kwon-Chung KJ, Juvvadi PR, Latgé JP, Steinbach WJ, Aspergillus fumigatus and related species, Cold Spring Harb. Perspect. Med 5 (2014) a019786, 10.1101/cshperspect.a019786. PMC4315914 25377144

[3] Juvvadi PR, Fortwendel JR, Rogg LE, Burns KA, Randell SH, Steinbach WJ, Localization and activity of the calcineurin catalytic and regulatory subunit complex at the septum is essential for hyphal elongation and proper septation in *Aspergillus fumigatus*, Mol. Microbiol 82 (2011) 1235–1259, 10.1111/j.1365-2958.2011.07886.x. PMC3225650 22066998

[4] Steinbach WJ, Cramer RA Jr., Perfect BZ, Asfaw YG, Sauer TC, Najvar LK, Kirkpatrick WR, Patterson TF, Benjamin DK Jr., Heitman J, Perfect JR, Calcineurin controls growth, morphology, and pathogenicity in *Aspergillus fumigatus*, Eukaryot. Cell 5 (2006) 1091–1103, 10.1128/ec.00139-06. PMC1489296 16835453

[5] Juvvadi PR, Fox D 3rd, Bobay BG, Hoy MJ, Gobeil SMC, Venters RA, Chang Z, Lin JJ, Averette AF, Cole DC, Barrington BC, Wheaton JD, Ciofani M, Trzoss M, Li X, Lee SC, Chen YL, Mutz M, Spicer LD, Schumacher MA, Heitman J, Steinbach WJ, Harnessing calcineurin-FK506-FKBP12 crystal structures from invasive fungal pathogens to develop antifungal agents, Nat. Commun. 10 (2019) 4275, 10.1038/s41467-019-12199-1. PMC6753081 31537789

[6] Juvvadi PR, Fortwendel JR, Pinchai N, Perfect BZ, Heitman J, Steinbach WJ, Calcineurin localizes to the hyphal septum in *Aspergillus fumigatus*: implications for septum formation and conidiophore development, Eukaryot. Cell 7 (2008) 1606–1610, 10.1128/ec.00200-08. PMC2547068 18606829

[7] Juvvadi PR, Pemble C.W.t., Ma Y, Steinbach WJ, Novel motif in calcineurin catalytic subunit is required for septal localization of calcineurin in *Aspergillus fumigatus*, FEBS Lett. 590 (2016) 501–508, 10.1002/1873-3468.12075. PMC4764407 26864964

[8] Juvvadi PR, Gehrke C, Fortwendel JR, Lamoth F, Soderblom EJ, Cook EC, Hast MA, Asfaw YG, Moseley MA, Creamer TP, Steinbach WJ, Phosphorylation of Calcineurin at a novel serine-proline rich region orchestrates hyphal growth and virulence in Aspergillus fumigatus, PLoS Pathog. 9 (2013) e1003564, 10.1371/journal.ppat.1003564. PMC3749960 23990785

[9] Pinchai N, Perfect BZ, Juvvadi PR, Fortwendel JR, Cramer RA Jr., Asfaw YG, Heitman J, Perfect JR, Steinbach WJ, *Aspergillus fumigatus* calcipressin CbpA is involved in hyphal growth and calcium homeostasis, Eukaryot. Cell 8 (2009) 511–519, 10.1128/ec.00336-08. PMC2669200 19252123

[10] Shwab EK, Juvvadi PR, Waitt G, Soderblom EJ, Barrington BC, Asfaw YG, Moseley MA, Steinbach WJ, Calcineurin-dependent dephosphorylation of the transcription factor CrzA at specific sites controls conidiation, stress tolerance, and virulence of *Aspergillus fumigatus*, Mol. Microbiol 112 (2019) 62–80, 10.1111/mmi.14254. PMC6615987 30927289

[11] Roh DH, Bowers B, Schmidt M, Cabib E, The septation apparatus, an autonomous system in budding yeast, Mol. Biol. Cell 13 (2002) 2747–2759, 10.1091/mbc.e02-03-0158. PMC117939 12181343

[12] Finger FP, One ring to bind them. Septins and actin assembly, Dev. Cell 3 (2002) 761–763, 10.1016/s1534-5807(02)00371-4. 12479801

[13] Pablo-Hernando ME, Arnaiz-Pita Y, Tachikawa H, del Rey F, Neiman AM, Vázquez de Aldana CR, Septins localize to microtubules during nutritional limitation in *Saccharomyces cerevisiae*, BMC Cell Biol. 9 (2008) 55, 10.1186/1471-2121-9-55. PMC2584027 18826657

[14] Silverman-Gavrila RV, Silverman-Gavrila LB, Septins: new microtubule interacting partners, Sci. World J 8 (2008) 611–620, 10.1100/tsw.2008.87. PMC5848705 18604445

[15] Juvvadi PR, Fortwendel JR, Rogg LE, Steinbach WJ, Differential localization patterns of septins during growth of the human fungal pathogen *Aspergillus fumigatus* reveal novel functions, Biochem. Biophys. Res. Commun 405 (2011) 238–243, 10.1016/j.bbrc.2011.01.017. PMC3045080 21219860

[16] Juvvadi PR, Belina D, Soderblom EJ, Moseley MA, Steinbach WJ, Filamentous fungal-specific septin AspE is phosphorylated in vivo and interacts with actin, tubulin and other septins in the human pathogen *Aspergillus fumigatus*, Biochem. Biophys. Res. Commun 431 (2013) 547–553, 10.1016/j.bbrc.2013.01.020. PMC3587984 23321313

[17] Vargas-Muñiz JM, Renshaw H, Waitt G, Soderblom EJ, Moseley MA, Palmer JM, Juvvadi PR, Keller NP, Steinbach WJ, Caspofungin exposure alters the core septin AspB interactome of *Aspergillus fumigatus*, Biochem. Biophys. Res. Commun 485 (2017) 221–226, 10.1016/j.bbrc.2017.02.116. PMC5384791 28238781

[18] Spiliotis ET, Nakos K, Cellular functions of actin- and microtubule-associated septins, Curr. Biol 31 (2021) R651–r666, 10.1016/j.cub.2021.03.064. PMC8194058 34033796

[19] Pope RE, Prade RA, Vesicle-driven endomembrane systems in fungi, Microbiol. Mol. Biol. Rev 90 (2026) e0029724, 10.1128/mmbr.00297-24. PMC13014759 41410470

[20] Vargas-Muñiz JM, Juvvadi PR, Steinbach WJ, Forging the ring: from fungal septins' divergent roles in morphology, septation and virulence to factors contributing to their assembly into higher order structures, Microbiology (Read.) 162 (2016) 1527–1534, 10.1099/mic.0.000359. PMC5068138 27559018

[21] Kinoshita M, Assembly of mammalian septins, J. Biochem 134 (2003) 491–496, 10.1093/jb/mvg182. 14607974

[22] Vargas-Muñiz JM, Renshaw H, Richards AD, Lamoth F, Soderblom EJ, Moseley MA, Juvvadi PR, Steinbach WJ, The *Aspergillus fumigatus* septins play pleiotropic roles in septation, conidiation, and cell wall stress, but are dispensable for virulence, Fungal Genet. Biol 81 (2015) 41–51, 10.1016/j.fgb.2015.05.014. PMC4519395 26051489

[23] Zekert N, Veith D, Fischer R, Interaction of the Aspergillus nidulans microtubule-organizing center (MTOC) component ApsB with gamma-tubulin and evidence for a role of a subclass of peroxisomes in the formation of septal MTOCs, Eukaryot. Cell 9 (2010) 795–805, 10.1128/ec.00058-10. PMC2863950 20348383

