# Supplementary material for: Calcineurin regulates septin cytoskeleton organization in the human fungal pathogen Aspergillus fumigatus
Source: Biochem Biophys Res Commun. Author manuscript; Available in PMC 2026 Nov 11. (PMC13752868; doi:10.1016/j.bbrc.2026.154773)
Supplement: Supplementary file 1 [file NIHMS2218993-supplement-Chris_et_al-Supplemental_Material.pdf]

Supplemental Information

Calcineurin Regulates Septin Cytoskeleton Organization in the Human Fungal Pathogen *Aspergillus fumigatus*

D. Christopher Cole[1¶], Erik J. Soderblom[2*], William J. Steinbach[3], Praveen R. Juvvadi[3*]

[1]Department of Pediatrics, Duke University School of Medicine. [2]Proteomics and Metabolomics Core Facility, Duke University School of Medicine. [3]Department of Pediatrics, Arkansas Children's Research Institute, University of Arkansas for Medical Sciences, Little Rock, AR, USA.

[¶]Present Address: Department of Biological Sciences, North Carolina State University, Raleigh, NC, USA.

Running Title: Calcineurin is required for proper septin organization

Key words: *Aspergillus fumigatus*, calcineurin, septins, actin, tubulin

***Corresponding Author:**

Praveen R. Juvvadi, Ph.D.
Department of Pediatrics
Arkansas Children's Research Institute
University of Arkansas for Medical Sciences
Little Rock, AR, USA
Email: prjuvvadi@uams.edu

Supplemental Figures

Supplemental Figure 1.

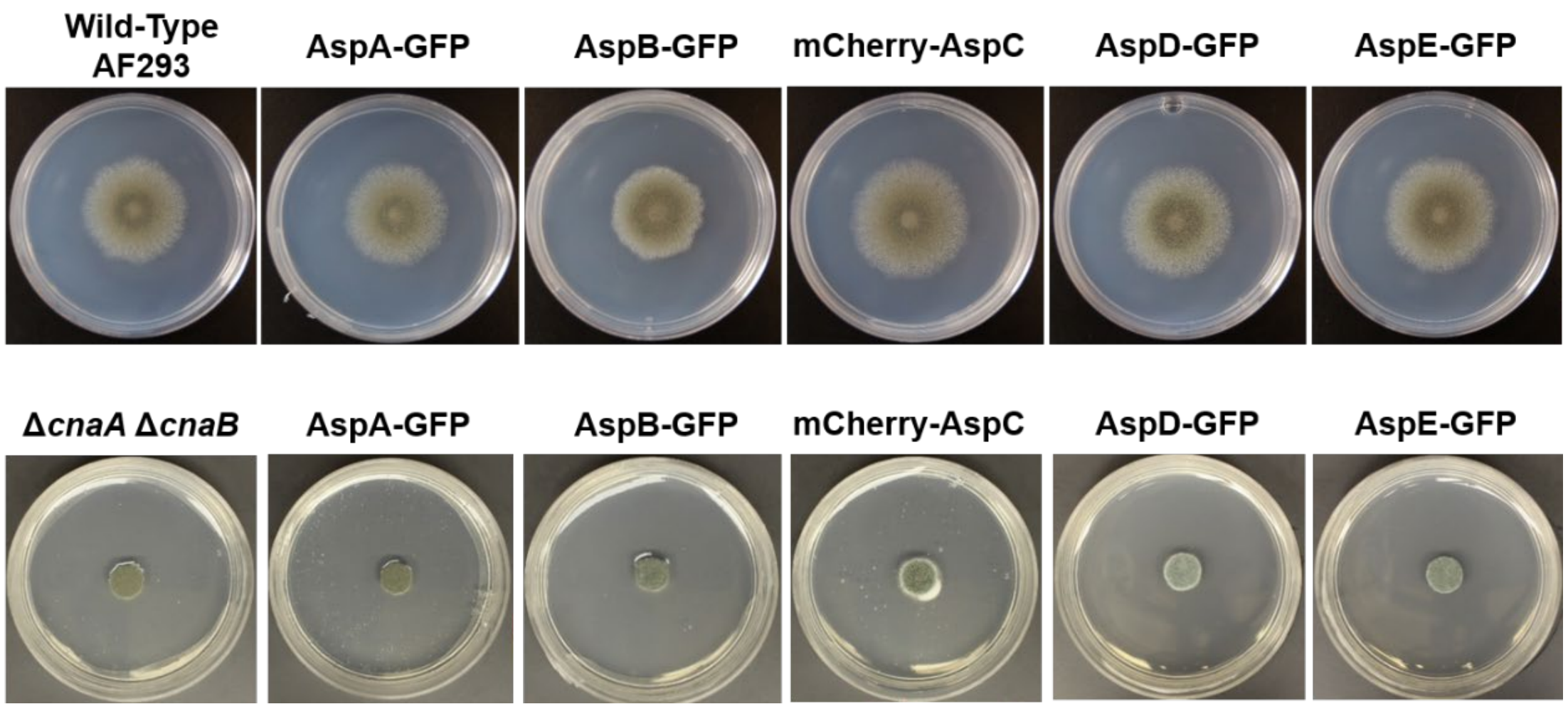

Suppl. Fig. 1. The wild type AF293 strain (10^6 spores/ml), the respective septin expression strains in the wild-type background, the calcineurin double mutant, and the respective septin expression strains in the calcineurin double mutant background were cultured on GMM agar media for 72 h at 37°C. Note that the expression of fluorescent tagged septins did not affect the growth of the various strains.
